# A Cross-Sectional Study of the Impact of ICU-Acquired Weakness: Prevalence, Associations, and Severity

**DOI:** 10.7759/cureus.49852

**Published:** 2023-12-02

**Authors:** Anas Khalil, Ruba A Alamri, Ghaida H Aljabri, Elham A Shahat, Rahaf I Almughamsi, Walaa A Almeshhen

**Affiliations:** 1 Internal Medicine, Taibah University, Medina, SAU; 2 Medicine and Surgery, Taibah University, Medina, SAU; 3 Medicine, Taibah University, Medina, SAU; 4 Intensive Care Unit, Taibah University, Medina, SAU

**Keywords:** mrc-sum score (mrc-ss), medical research council (mrc) scale for muscle strength, muscle weakness, intensive care unit-acquired weakness (icu-aw), intensive care unit (icu)

## Abstract

Background and objective

ICU-acquired weakness (ICU-AW) refers to a group of neuromuscular lesions that can develop in the ICU. It leads to decreased physical function, increased in-ICU and in-hospital mortality, and increased healthcare costs. Given its high prevalence and significant impact on patient outcomes, it is essential to have a deeper understanding of ICU-AW. In light of this, this study aimed to ascertain the prevalence, associations, and severity of ICU-AW at a tertiary hospital in the Kingdom of Saudi Arabia (KSA) and to evaluate physician awareness of this condition.

Methods

A cross-sectional study was conducted in the ICU of Al Madina General Hospital, Medina, KSA, from April 22 to August 22, 2022, involving patients who were 18 years or older and met the inclusion criteria (n=101). The overall muscle strength was assessed daily by using the Medical Research Council (MRC) scale for muscle strength. ICU-AW was identified in patients who experienced a decline in their MRC-Sum Score (MRC-SS) during their ICU stay.

Results

A total of 101 patients were enrolled in the study. The incidence of ICU-AW was 16.8% (n=17), with 23.5% exhibiting significant weakness and 76.5% having severe weakness. Post hoc comparisons showed that females had a higher incidence of ICU-AW. Fisher's exact test revealed a statistically significant relationship between ICU-AW and the longer duration of ICU stay (p=0.001), use of mechanical ventilation (p=0.034), and low hemoglobin levels (p=0.037).

Conclusions

ICU-AW was observed in 16.8% (n=17) of patients in our cohort, highlighting the significance of this condition. The study revealed a noteworthy correlation between ICU-AW and female sex, extended ICU stays, mechanical ventilation, and anemia.

## Introduction

ICU-acquired weakness (ICU-AW) comprises a collection of neuromuscular impairments that can arise as a secondary complication during the treatment of other critical conditions in the ICU [1]. This condition typically manifests as symmetrical weakness in the limbs (with proximal muscles more affected than distal ones) and respiratory muscles, while facial and ocular muscles remain unaffected [2,3]. ICU-AW is most commonly caused by critical illness polyneuropathy (CIP), critical illness myopathy (CIM), or a combination of the two, known as critical illness polyneuromyopathy (CIPNM). While these conditions may present with similar clinical symptoms, their underlying pathophysiology varies. While the precise pathophysiology of ICU-AW is not fully understood, it involves complex structural and functional changes within myofibers and neurons [1]. CIP is an axonal sensorimotor polyneuropathy characterized by the loss of individual nerve fibers, whereas weakness in CIM results from the loss of thick myofilaments and subsequent myofiber death in skeletal muscle without a neurogenic etiology [4].

Muscle weakness is a common issue encountered in the ICU, with a global incidence rate ranging from 25 to 85%. Moreover, up to 36% of patients may continue to experience muscle weakness even after their discharge from the ICU [5]. Several risk factors for developing ICU-AW have been identified, including both non-modifiable and modifiable factors. Non-modifiable risk factors include prolonged critical illness, such as sepsis and inflammation, multiple organ failure, and extended periods of mechanical ventilation and ICU stay [6-10]. Modifiable risk factors include elevated blood lactate levels, hyperglycemia [10], and certain medications such as vasoactive agents, sedatives, corticosteroids, and neuromuscular blocking agents [8,10-13], as well as extended periods of bed rest and immobilization [12,14]. Additionally, women and older patients are at a higher risk of developing weakness than men and younger patients [6]. Premorbid disability and frailty may also increase the severity of weakness, while premorbid obesity is an independent protective factor against the development of ICU-AW and muscle atrophy [15].

The Medical Research Council (MRC) scale for muscle strength is currently the most widely used tool for evaluating and diagnosing ICU-AW, despite its major limitation of requiring patients to be awake and cooperative [2,16-18]. Other modalities are also being used to assess and diagnose ICU-AW. Electrophysiological assessments can be used as an alternative to the MRC scale among uncooperative patients. Ultrasonography is another option for quick and repeated bedside evaluation of muscle quantity and quality, but it may underestimate muscle and protein loss [3,19-21]. CT and MRI are more accurate and reliable in detecting muscle infiltration by adipose tissue and quantifying fat-free muscle mass, but they are expensive, require specialized staff and software, and are logistically challenging [19,21]. Additionally, CT exposes patients to a high level of radiation. Lastly, performing a nerve or muscle tissue biopsy is rare due to its invasive nature and it is not a standard procedure in routine clinical practice.

ICU-AW is associated with numerous unfavorable short- and long-term consequences, such as increased in-ICU and in-hospital mortality, prolonged mechanical ventilation and hospitalization, elevated healthcare costs, a greater likelihood of extended rehabilitation care, and decreased physical function and quality of life in the long run [1]. Given its high prevalence and significant impact on patient outcomes, it is essential to have a deeper understanding of ICU-AW. However, despite being a prevalent issue with serious implications, there is a lack of local research on its prevalence, diagnosis, risk factors, and treatment. Since there are no specific drugs or treatments available for ICU-AW, the treatment primarily focuses on nutrition and supportive therapies to alleviate symptoms. Therefore, it is crucial to investigate the pathophysiological mechanisms of ICU-AW and identify specific therapeutic drugs and strategies to improve patient outcomes [22].

Objectives

The objectives of this study were as follows: to ascertain the prevalence and severity of ICU-AW in a tertiary hospital located in Saudi Arabia, to explore various associations linked to ICU-AW, and to evaluate the level of physician awareness and recognition of this condition.

## Materials and methods

Study design

This was a single-center cross-sectional study approved by the ethics committee of Taibah University and the General Directorate of Health Affairs of Medina-Ministry Of Health (MOH).

Subjects and procedures

The study included all patients who were admitted to the ICU of Al Madina General Hospital between April 22 to August 22, 2022, who were 18 years of age or older and met the inclusion criteria.

Within 48 hours of ICU admission, a total of 141 patients were consecutively screened for eligibility. Inclusion criteria consisted of patients aged 18 years or older who were admitted to the ICU (N=101). Patients who were discharged from the ICU within 48 hours, those under the age of 18 years, and those with a condition or neurological diseases severely limiting mobility were excluded from the study (n=40). 

The overall muscle strength was assessed using the MRC scale for muscle strength, which is a reliable and widely used method for identifying ICU-AW in critically ill patients (Table 1) [23-25]. The MRC system evaluates muscle strength in six categories: wrist extension, elbow flexion, bilateral shoulder abduction, hip flexion, knee extension, and foot dorsiflexion. Each group is given a score from 0 to 5 based on the patient's ability to move against gravity and resistance. The total score ranges from 0 to 60, with higher scores indicating greater muscle strength. Patients who score less than 48 are identified as having significant muscle weakness, and those who score less than 36 are identified as having severe weakness. We characterized patients as having ICU-AW if their MRC-Sum Score (MRC-SS) decreased to a value below 48 at any time during their ICU stay in comparison to the initial recorded MRC-SS. Those who had an initial MRC-SS of less than 48 but did not experience a decrease during their ICU stay were characterized to have muscle weakness not related to ICU-AW. The data of the MRC-SS was obtained on the 3rd, 7th, 14th, 21st, and 28th days of admission. MRC-SS data was not obtained beyond the 28th day of the patient’s admission to the ICU.

**Table 1:** Medical Research Council (MRC) scale for muscle strength* *[26]

Score	Interpretation
0	No contraction
1	Contraction without movement
2	Movement with gravity eliminated
3	Movement against gravity
4	Movement against resistance
5	Normal muscle force

The researchers collected data on various sociodemographic and clinical variables, such as gender, age, duration of ICU stay, use of mechanical ventilation, medications, laboratory results, evidence of weakness, and patient comorbidities. The contents of the data collection sheet can be found in the Appendices section.

Statistical analysis

The SPSS Statistics version 26.0 (IBM Corp., Armonk, NY) was used for data analysis. Data normality was tested using the Kolmogorov-Smirnov test. Continuous data were presented as median and interquartile range (IQR), while categorical variables were represented as frequencies and percentages. Categorical variable analysis was performed using the Chi-square test, and Fisher’s exact test was used to determine if there was a significant association between ICU-AW and the sociodemographics of the sample, the duration of the ICU stay, use of mechanical ventilation, laboratory results, medications, evidence of weakness, and patient comorbidities. A p-value of less than 0.05 was considered statistically significant, and the confidence interval was set at 95%.

Ethical considerations

The study received approval from the ethics committees of Taibah University, Al Medina General Hospital, and the General Directorate of Health Affairs of Medina-Ministry Of Health (MOH). An official letter explaining the study's purpose and setting was drafted before conducting the study. Each patient participating in the study or their healthcare proxy was counseled about the study purpose and process and informed about their right to refuse participation in the study, but written consent to participate in the study was not obtained.

## Results

In this study, we initially screened a total of 141 patients, of which 40 did not meet the inclusion criteria and hence were excluded. Thus, the final sample size consisted of 101 patients; Table 2 presents their sociodemographic characteristics. Of the included patients, 51.5% (n=52) were male, and 48.5% (n=49) were female, with a median age of 67 years (IQR: 23) and a median ICU stay of nine days (IQR: 11). Mechanical ventilation was required for 41.6% of the patients, and sedatives were given to 27.7%, while 60.4% of the patients received steroids, and only 5% were given paralytics. Muscle weakness was observed in 50.5% of the included patients (both ICU-AW and non-ICU-AW). Evidence of ICU-AW was found in 16.8% (N=17) of the included patients. Of those patients, 23.5% had significant weakness and 76.5% had a severe weakness.

**Table 2:** Prevalence of ICU-AW and sociodemographic and clinical characteristics of the sample (n=101) IQR: interquartile Range; ICU: intensive care unit; ICU-AW: intensive care unit–acquired weakness

Characteristics	Frequency	Percentage
Study sample		
Inclusion	101	71.6%
Exclusion	40	28.4%
Gender		
Male	52	51.5%
Female	49	48.5%
Age, years, median (IQR)	67 (23)	
Duration of ICU stay, days, median (IQR)	9 (11)	
Mechanical ventilation		
No	59	58.4%
Yes	42	41.6%
Medications		
Sedatives	28	27.7%
Steroids	61	60.4%
Paralytics	5	5%
Evidence of weakness (ICU-AW + weakness not due to ICU-AW)		
No	50	49.5%
Yes	51	50.5%
ICU-acquired weakness		
No	84	83.2%
Yes	17	16.8%
Grade of ICU-AW		
Significant weakness	4	4.0%
Severe weakness	13	12.9%

**Figure 1:**
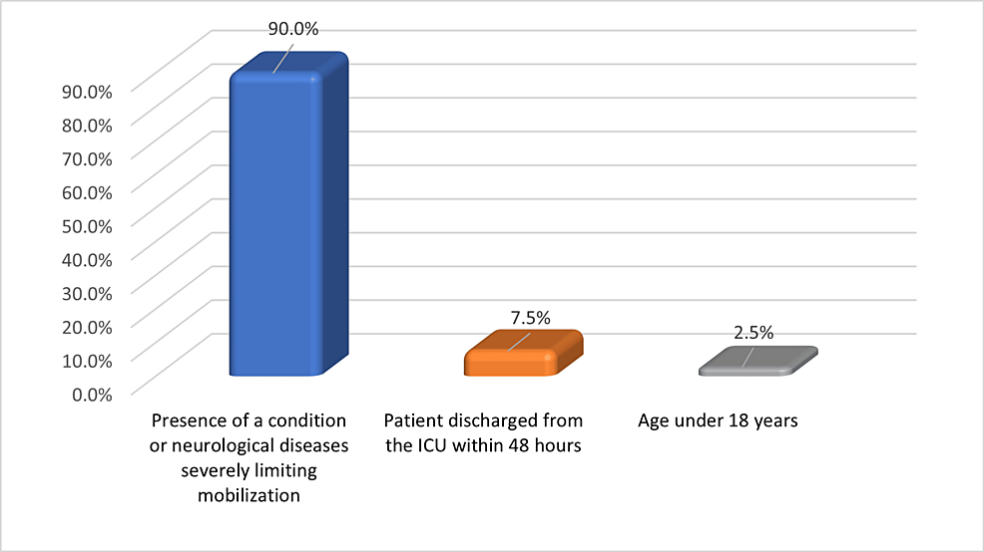
Reasons for excluding patients from the study (n=40) ICU: intensive care unit

Figure 1 illustrates the reasons for excluding patients from the study; the "presence of a condition or neurological diseases severely limiting mobilization" was the most common cause (90%), followed by "patients discharged from the ICU within 48 hours" (7.5%). 

**Figure 2:**
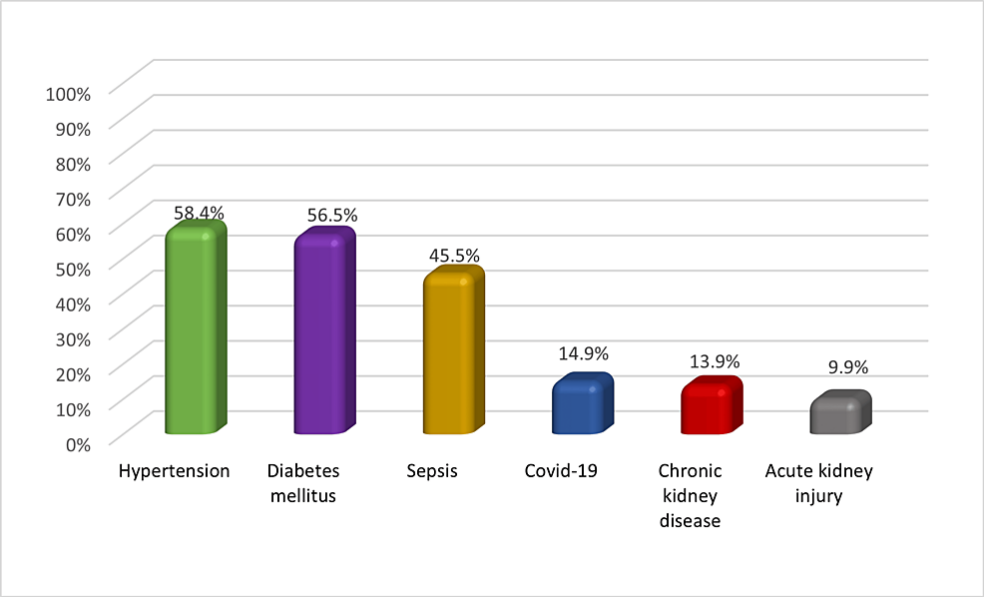
Distribution of sample by patient comorbidities (n=101) COVID-19: coronavirus disease 2019

Figure 2 depicts the breakdown of the study sample based on the presence of comorbidities among patients. The data revealed that 58.4% of the patients had hypertension, while 56.5% had diabetes mellitus, and 45.5% had sepsis. In contrast, only 9.9% of patients suffered from acute kidney injury.

Table 3 presents the Person's Chi-squared test, which indicated a significant association between ICU-AW and patients' gender (p=0.046). Post hoc comparisons demonstrated that females had a higher incidence of ICU-AW compared to males. Additionally, Fisher's exact test revealed a statistically significant relationship between ICU-AW and the duration of ICU stay (p=0.001), with post hoc comparisons showing that patients who stayed in the ICU for 14-28 days had a higher incidence of ICU-AW than those who stayed for 7-14 days. Furthermore, there was a significant association between ICU-AW and the need for mechanical ventilation (p=0.034). 

**Table 3:** Association between ICU-AW and sociodemographic of the sample, clinical characteristics, and patient comorbidities (n=101) ^a^Fisher's exact test. *P-value is statistically significant ICU: intensive care unit; ICU-AW: intensive care unit–acquired weakness

Factors	Categories	ICU-AW		P-value
		No	Yes	
Sex	Male	47 (56%)	5 (29.4%)	0.046*
	Female	37 (44%)	12 (70.6%)	
Age^a^	Less than 40 years	11 (13.1%)	3 (17.6%)	0.710
	40-50 years	8 (9.5%)	0 (0%)	
	51-60 years	13 (15.5%)	3 (17.6%)	
	More than 60 years	52 (61.9%)	11 (64.7%)	
Duration of ICU stay (days)^a^	Less than 7 days	33 (39.3%)	0 (0%)	0.001*
	7-14 days	31 (36.9%)	7 (41.2%)	
	14-28 days	20 (23.8%)	10 (58.8%)	
Mechanical ventilation	No	53 (63.1%)	6 (35.3%)	0.034*
	Yes	31 (36.9%)	11 (64.7%)	
Diabetes mellitus	No	45 (53.6%)	9 (52.9%)	0.962
	Yes	39 (46.4%)	8 (47.1%)	
Hypertension	No	35 (41.7%)	7 (41.2%)	0.970
	Yes	49 (58.3%)	10 (58.8%)	
Acute kidney injury^a^	No	75 (89.3%)	16 (94.1%)	0.469
	Yes	9 (10.7%)	1 (5.9%)	
Chronic kidney disease^a^	No	73(86.9%)	14 (82.4%)	0.700
	Yes	11 (13.1%)	3 (17.6%)	
Covid-19^a^	No	71 (84.5%)	15 (88.2%)	.517
	Yes	13 (15.5%)	2 (11.8%)	
Sepsis	No	46 (54.8%)	9 (52.9%)	0.891
	Yes	38 (45.2%)	8 (47.1%)	
Sedatives	No	62 (73.8%)	11 (64.7%)	0.553
	Yes	22 (26.2%)	6 (35.3%)	
Steroids	No	33 (39.3%)	7 (41.2%)	0.884
	Yes	51 (60.7%)	10 (58.8%)	
Paralytics^a^	No	80 (95.2%)	16 (94.1%)	0.610
	Yes	4 (4.8%)	1 (5.9%)	

As presented in Table 4, Fisher's exact test showed that there was a statistically significant association between ICU-AW and hemoglobin levels in the blood (p=0.037). Post hoc comparisons revealed that patients who had anemia had a higher rate of ICU-AW compared to patients whose hemoglobin was in the normal range.

**Table 4:** Association between ICU-AW and various laboratory tests (n=101) ^a^Fisher's exact test. *P-value is statistically significant ICU: intensive care unit; ICU-AW: intensive care unit–acquired weakness

Laboratory tests	Categories	ICU-AW, n (%)		P-value
		No	Yes	
Hemoglobin^a^	Normal	18 (21.4%)	0 (0%)	0.037*
	Anemia	66 (78.6%)	17 (100%)	
Platelets	Normal	65 (77.4%)	11 (64.7%)	0.269
	Thrombocytopenia	19 (22.6%)	6 (35.3%)	
Lactate	Normal	62 (73.8%)	10 (58.8%)	0.213
	High lactate	22 (26.2%)	7 (41.2%)	
Creatinine kinase^a^	Normal	73 (86.9%)	17 (100%)	0.203
	High creatinine kinase level	11 (13.1%)	0 (0%)	
Glucose	Normal	35 (41.7%)	7 (41.2%)	0.984
	Hyperglycemia	23 (27.4%)	5 (29.4%)	
	Uncontrolled hyperglycemia	26 (31%)	5 (29.4%)	
Albumin^a^	Normal	38 (45.2%)	4 (23.5%)	0.113
	Hypoalbuminemia	46 (54.8%)	13 (76.5%)	
Calcium	Normal	33 (39.3%)	8 (47.1%)	0.552
	Hypocalcemia	51 (60.7%)	9 (52.9%)	
Potassium	Normal	63 (75%)	12 (70.6%)	0.704
	Hypokalemia	21 (25%)	5 (29.4%)	

A comprehensive review of the medical charts revealed that none of the patients identified with ICU-AW in our study had received a formal diagnosis of the condition from the treating team, as evidenced by the absence of any mention of ICU-AW in the medical charts.

## Discussion

In this cross-sectional study, we observed that the prevalence of ICU-AW was 16.8% (n=17), with 76.5% of cases classified as severe ICU-AW. Previous studies have reported a broad range in terms of ICU-AW prevalence (25-85%), which can be attributed to the lack of universal consensus regarding its definition and the varied tools employed to assess ICU-AW. We defined ICU-AW if the patient met the following two criteria: a decrease in MRC-SS from the initial recorded MRC-SS and a decline in MRC-SS to a value of less than 48. This likely led to a more precise detection of ICU-AW but also to a lower prevalence of the disease. Furthermore, muscle weakness was identified in approximately half of the patients included in the study (50.5%), encompassing both those diagnosed with ICU-AW and those exhibiting muscle weakness that did not meet the criteria mentioned above.

Other findings of the study revealed a statistically significant correlation between ICU-AW and female sex, extended ICU stay, use of mechanical ventilation, and anemia. The correlation between ICU-AW and ICU length of stay and use of mechanical ventilation is consistent with findings of other studies conducted in KSA and China [27,28]. However, the study by Asfour found no difference in ICU-AW between males and females [27]. 

This study showed no significant difference in the incidence of ICU-AW and the following comorbidities and lab values: COVID-19, sepsis, diabetes mellitus, hypertension, chronic kidney disease, acute kidney injury, and albumin level. This is in contrast to many studies that found that COVID-19 infection is associated with a significantly higher incidence of ICU-AW [29-31]. Furthermore, Yang et al. [10] conducted a systemic review and meta-analysis of 14 studies that aimed to determine the risk factors for ICU-AW. The results showed that both Systemic Inflammatory Response Syndrome (SIRS) and sepsis were independent risk factors for ICU-AW, especially if they lasted for a long duration of time.

Our study did not show a significant association between ICU-AW and the use of sedatives, neuromuscular blocking agents (NMBAs), or corticosteroids; this contrasts with other studies that suggest an association between medications commonly used in ICU and ICU-AW. Latronico et al. found that immobility caused by prolonged sedation increases the risk of ICU-AW [2]. A study by Qin et al. has shown that prolonged corticosteroid use can result in steroid myopathies and may contribute to ICU-AW by suppressing muscle protein synthesis, which causes muscle wasting [32]. Furthermore, a meta-analysis of systemic corticosteroid administration and ICU-AW concluded that corticosteroid use was a significant risk factor for developing ICU-AW and that reducing corticosteroids would help lower ICU-AW [33]. Yang et al. found that NMBAs will cause adverse muscle effects that increase the probability of muscle atrophy and exacerbate ventilator-induced diaphragmatic weakness [10].

Of note, although our study revealed an ICU-AW prevalence of 16.8% (N=17) among patients, none of them had an officially documented diagnosis of the condition in their medical charts as confirmed by the treating team. This finding highlights a significant gap in patient management, with a lack of proactive measures to prevent or address this condition.

Our study has some limitations, which include its cross-sectional design, the fact that it excluded a large number of patients, its single-center design, and its limited sample size. The study used the MRC scale for muscle strength to assess ICU-AW, which is currently the most widely used and reliable tool for evaluating and diagnosing ICU-AW, despite its major limitations [2,16-18].

## Conclusions

The prevalence of ICU-AW in this study was 16.8% (n=17) while muscle weakness either due to ICU-AW or non-ICU-AW was found in 50.5% of the study population. However, none of these patients had an officially documented diagnosis of the condition in their medical charts prepared by the treating team. This finding highlights a significant gap in patient management, with a lack of proactive measures taken to prevent or address this condition. The study also revealed a noteworthy correlation between ICU-AW and female sex, extended ICU stays, mechanical ventilation, and anemia.

## Appendices

Data collection sheet

A Cross-Sectional Study on the Impact of ICU-Acquired Weakness: Prevalence, Associations and Severity

Participant number: …………………….                                                        Date: ……/……/…… 

This is a data collection form for the “A Cross-Sectional Study on the Impact of ICU-Acquired Weakness: Prevalence, Associations and Severity “. To be filled by the study investigators and team.

Patient eligibility

In order for the subject to be eligible for the study, he/she must meet ALL inclusion criteria and none of the exclusion criteria. If the response is “No” to any of the inclusion criteria, the subject is not eligible to participate in the study.

Inclusion criteria

All male and female patients aged 18 years and older who were admitted to the ICU at the time of signing the  consent form: 

□       Yes              □ No

Exclusion criteria

1.     Patients discharged from the ICU within 48 hours:

□       Yes              □ No

2.     Age under 18 years:

□       Yes              □ No

3.     Presence of a condition or neurological disease severely limiting mobilization:

□       Yes              □ No

A.  Baseline demographic data

1.       Patient name: ……………………………………

2.       File number: …………………………………….

3.       Gender:        □ Male       □ Female

4.       Age: ……………years

5.       Diagnosis: ……………………………………….

B.   Comorbidities

o   Diabetes.

o   Hypertension.

o   Acute kidney injury.

o   Chronic kidney disease.

o   Covid-19.

o   Sepsis.

o   Others: …………………………………………

o   None.

C.   Duration of ICU stay

Number of days ………….

D.   Application of assisted mechanical ventilation

□       Yes              □ No

□       Date of intubation:     /     / 

□       Date of extubation:     /    /      

□       Duration of mechanical ventilation: ……………

**Table 5:** MRC score results

Day of MRC score	0	1	2	3	4	5
ICU Admission (Day1) Date: / /2022						
Day (2) Date: / /2022						
Day (3) Date: / /2022						
Day (4) Date: / /2022						
Day (5) Date: / /2022						
Day (6) Date: / /2022						
Day (7) Date: / /2022						
Day (8) Date: / /2022						
Day (9) Date: / /2022						
Day (10) Date: / /2022						
Day (11) Date: / /2022						
Day (12) Date: / /2022						
Day (13) Date: / /2022						
Day (14) Date: / /2022						
Day (15) Date: / /2022						
Day (16) Date: / /2022						
Day (17) Date: / /2022						
Day (18) Date: / /2022						
Day (19) Date: / /2022						
Day (20) Date: / /2022						
Day (21) Date: / /2022						
Day (22) Date: / /2022						
Day (23) Date: / /2022						
Day (24) Date: / /2022						
Day (25) Date: / /2022						
Day (26) Date: / /2022						
Day (27) Date: / /2022						
Day (28) Date: / /2022						

**Table 6:** Medication regimen

Day of medicine administration	Sedative	Steroids	Paralytics
Day (1)	□ Yes □ No	□ Yes □ No	□ Yes □ No
Day (2)	□ Yes □ No	□ Yes □ No	□ Yes □ No
Day (3)	□ Yes □ No	□ Yes □ No	□ Yes □ No
Day (4)	□ Yes □ No	□ Yes □ No	□ Yes □ No
Day (5)	□ Yes □ No	□ Yes □ No	□ Yes □ No
Day (6)	□ Yes □ No	□ Yes □ No	□ Yes □ No
Day (7)	□ Yes □ No	□ Yes □ No	□ Yes □ No
Day (8)	□ Yes □ No	□ Yes □ No	□ Yes □ No
Day (9)	□ Yes □ No	□ Yes □ No	□ Yes □ No
Day (10)	□ Yes □ No	□ Yes □ No	□ Yes □ No
Day (11)	□ Yes □ No	□ Yes □ No	□ Yes □ No
Day (12)	□ Yes □ No	□ Yes □ No	□ Yes □ No
Day (13)	□ Yes □ No	□ Yes □ No	□ Yes □ No
Day (14)	□ Yes □ No	□ Yes □ No	□ Yes □ No
Day (15)	□ Yes □ No	□ Yes □ No	□ Yes □ No
Day (16)	□ Yes □ No	□ Yes □ No	□ Yes □ No
Day (17)	□ Yes □ No	□ Yes □ No	□ Yes □ No
Day (18)	□ Yes □ No	□ Yes □ No	□ Yes □ No
Day (19)	□ Yes □ No	□ Yes □ No	□ Yes □ No
Day (20)	□ Yes □ No	□ Yes □ No	□ Yes □ No
Day (21)	□ Yes □ No	□ Yes □ No	□ Yes □ No
Day (22)	□ Yes □ No	□ Yes □ No	□ Yes □ No
Day (23)	□ Yes □ No	□ Yes □ No	□ Yes □ No
Day (24)	□ Yes □ No	□ Yes □ No	□ Yes □ No
Day (25)	□ Yes □ No	□ Yes □ No	□ Yes □ No
Day (26)	□ Yes □ No	□ Yes □ No	□ Yes □ No
Day (27)	□ Yes □ No	□ Yes □ No	□ Yes □ No
Day (28)	□ Yes □ No	□ Yes □ No	□ Yes □ No

**Table 7:** Baseline laboratory investigations

Laboratory tests	Result
Hemoglobin	
Platelets	
Lactate	
Creatinine kinase CK	
Blood glucose	
Albumin	
Calcium Ca	
Potassium K	

Data collector                                                  Investigator agreement

                                                                (PI/co-PI/designee)

Name: ……………………………                          Name: ………………………………………

I.D: ………………………………                             I.D:………………………………………….

Position: …………………………                          Position: ……………………………………

Signature: ………………….……                         Signature: …………………………………..

Date: …………………………….                           Date: ………………………………………
